# ﻿Revision of the orb-weaver spider genus *Gea* C.L. Koch, 1843 (Araneae, Araneidae) from China

**DOI:** 10.3897/zookeys.1191.117592

**Published:** 2024-02-09

**Authors:** Xiaoqi Mi, Feng Liu, Cheng Wang, Jiahui Gan, Yibei Wu

**Affiliations:** 1 College of Agriculture and Forestry Engineering and Planning, Guizhou Provincial Key Laboratory of Biodiversity Conservation and Utilization in the Fanjing Mountain Region, Tongren University, Tongren 554300, Guizhou, China Tongren University Tongren China; 2 Guangdong University of Petrochemical Technology, Maoming 525000, Guangdong, China Guangdong University of Petrochemical Technology Maoming China

**Keywords:** Arachnida, Argiopinae, diagnosis, morphology, new species, taxonomy

## Abstract

The orb-weaver spider genus *Gea* C.L. Koch, 1843 from China is revised, and three species including one new species, are recognized: *Geajingdong* Mi, Wang & Gan, **sp. nov.** (♂♀) from Yunnan; *Geaspinipes* C.L. Koch, 1843 (♂♀) from Guangdong, Guangxi, Guizhou, Hainan, Taiwan, and Yunnan; and *Geasubarmata* Thorell, 1890 (♂♀) from Guangxi and Hainan. *Geasubarmata* is newly recorded in China.

## ﻿Introduction

The orb-weaver spider subfamily Argiopinae consists of three genera, *Gea* C.L. Koch, 1843, *Argiope* Audouin, 1826, and *Neogea* Levi, 1983 (Levi 1983). This subfamily differs from other araneid subfamilies in having the posterior eye row procurved in dorsal view, and it is also characterized by sexual dimorphism (Levi 1983). The subfamily Argiopinae of the Western Pacific region has been revised by Levi (1983), who included in it 49 *Argiope* species, seven *Gea* species, and two *Neogea* species; eight species of *Argiope* occur in China, but no species of *Gea* were known from China.

The genus *Gea* contains 13 species and subspecies, which are mainly distributed in Africa, Asia, and Australia, and *Geaheptagon* (Hentz, 1850) is introduced to the USA to Argentina (WSC 2024). *Geaspinipes* has been almost concurrently reported from Guizhou and Yunnan (Yin et al. 1997) and Taiwan (Chang and Chang 1997) and is the only known *Gea* species known from China at present (Song et al. 1999; WSC 2024).

The *Gea* specimens collected in China were examined, and three species including a new species, are identified. They are described in this paper.

## ﻿Material and methods

All specimens were collected by beating shrubs or by hand and are preserved in 75% ethanol. The specimens are deposited in the Museum of Tongren University, China (**TRU**). Methods follow Mi et al. (2023).

All measurements are given in millimeters. Leg measurements are given as total length (femur, patella + tibia, metatarsus, tarsus). Abbreviations used in the text and figures are as follows: **ALE** anterior lateral eye; **AME** anterior median eye; **C** conductor; **CD** copulatory duct; **CO** copulatory opening; **E** embolus; **FD** fertilization duct; **LP** lateral plate; **MA** median apophysis; **MOA** median ocular area; **PLE** posterior lateral eye; **PME** posterior median eye; **Sp** spermatheca.

## ﻿Taxonomy

### ﻿Family Araneidae Clerck, 1757

#### ﻿Genus Gea

Taxon classificationAnimaliaAraneaeAraneidae

C.L. Koch, 1843

154165E8-ED30-5857-A684-268F8714074F

Gea C.L. Koch, 1843: 101.

##### Type species.

*Geaspinipes* C.L. Koch, 1843.

##### Diagnosis.

*Gea* is distinguished from *Argiope* by having the posterior eyes about equally spaced, while *Argiope* has the posterior median eyes farther from the posterior lateral eyes than the posterior median eyes from each other (Levi 1983: figs 27, 45, 64). *Gea* differs from *Neogea* in having the cephalic region behind the eyes not swollen, while in *Neogea* this region of the head is swollen (Levi 1983: figs 290, 292).

##### Description.

Small to medium-sized spiders with female total length of 3.65–9.00 mm and male total length of 3.00–4.30 mm. Carapace pear-shaped, yellow to yellowish brown. Legs yellow to yellowish brown, always with dark annuli; coxa I of male without hook; femur II of male without groove; tibia II of male not expanded. Abdomen shield-shaped dorsal often with a pair of low anterolateral humps in females, pale with a pair of dark patches close to humps and dark folium posteriorly or dark with white spots. Ventral abdomen pale with irregular dark patches or white spots.

***Pedipalp*** of male without basal femoral protrusion; patella with only one bristle; paracybium fingerlike or flattened fingerlike; median apophysis bifurcated; dorsal ramus often weaker than ventral ramus; embolus extremely long and curved, thick at base, tapering to filiform end; conductor broad, curved, wrapped distal part of embolus.

***Epigynum*** weakly sclerotized; median septum separating two depressions; copulatory openings situated on edges of depressions; copulatory ducts twisted, a bit longer than spermatheca; spermathecae elongate kidney-shaped, S-shaped, or bean-shaped, either touching or not.

##### Comment.

Spination of femur I is not useful to characterize these *Gea* species.

#### ﻿ Gea jingdong

Taxon classificationAnimaliaAraneaeAraneidae

Mi, Wang & Gan sp. nov.

BCCE37D0-16B9-5C7A-81F7-6D3B9850A3AB

https://zoobank.org/64B67A05-C6F7-419B-B2A2-EE672A6E4BE7

**Figure 1.**
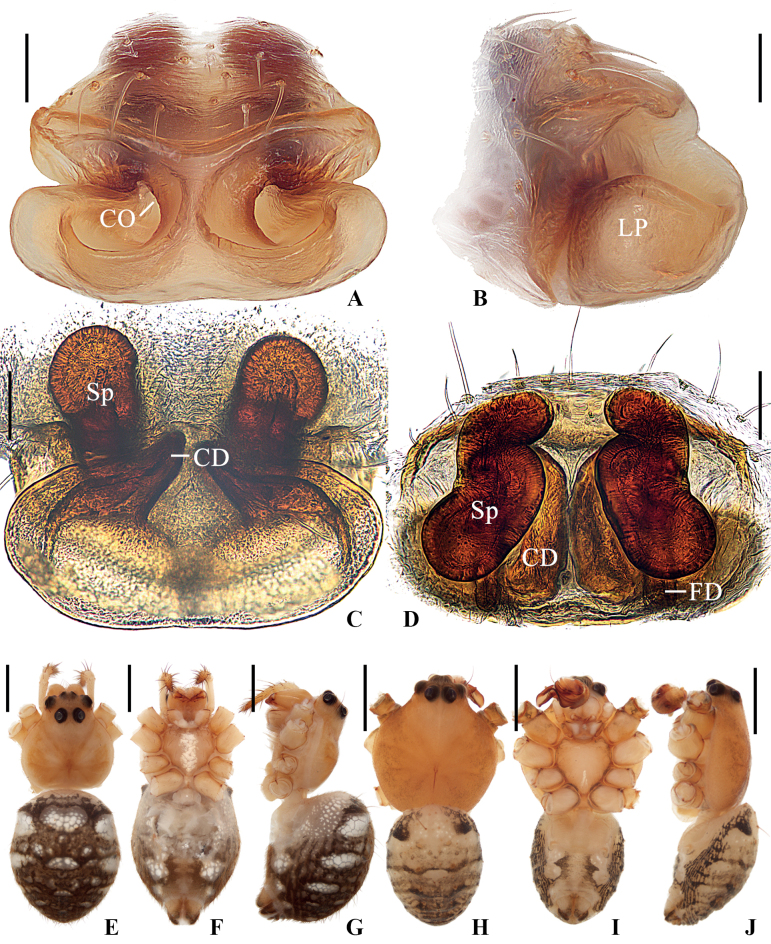
*Geajingdong* Mi, Wang & Gan, sp. nov. **A–G** female paratype TRU-Araneidae-269 **H–J** male holotype **A** epigyne, ventral view **B** ibid., lateral view **C** vulva, posterior view **D** ibid., dorsal view **E, H** habitus, dorsal view **F, I** ibid., ventral view **G, J** ibid., lateral view. Scale bars: 0.1 mm (**A–D**); 1 mm (**E–J**). Abbreviations: CD copulatory duct, CO copulatory opening, FD fertilization duct, LP lateral plate, Sp spermatheca.

**Figure 2.**
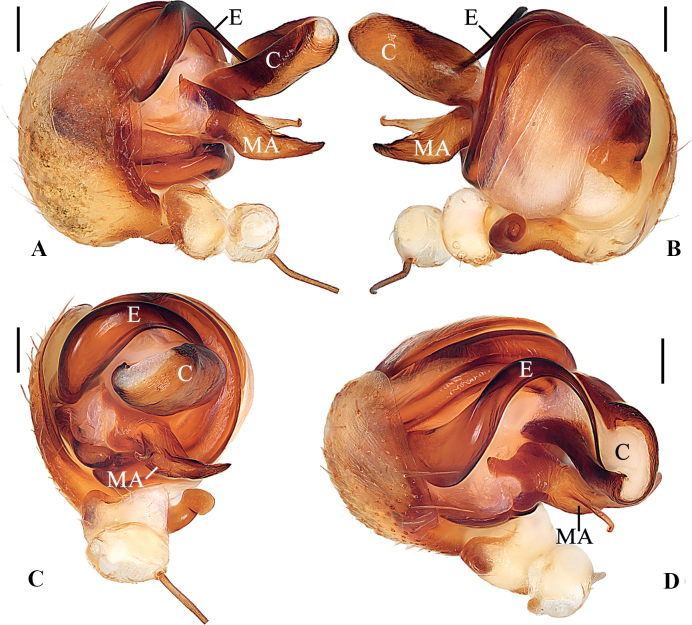
*Geajingdong* Mi, Wang & Gan, sp. nov. male holotype **A** pedipalp, prolateral view **B** ibid., retrolateral view **C** ibid., ventral view **D** ibid., apical view. Scale bars: 0.1 mm. Abbreviations: C conductor, E embolus, MA median apophysis.

Figs 1 , 2 , 7A–D , 8

##### Type materials.

***Holotype***: China • ♂; Yunnan Province, Dali Bai Autonomous Prefecture, Jingdong Yi Autonomous County, Jinping Township, Yubishan Park; 24°27.01'N, 101°49.53'E; ca 1270 m elev.; 16.VIII.2015; X.Q. Mi et al. leg.; TRU-Araneidae-268. ***Paratypes***: 3♀♀; same data as for holotype; TRU-Araneidae-269–271.

##### Etymology.

The specific name is a noun in apposition and refers to the type locality.

##### Diagnosis.

**Figure 3.**
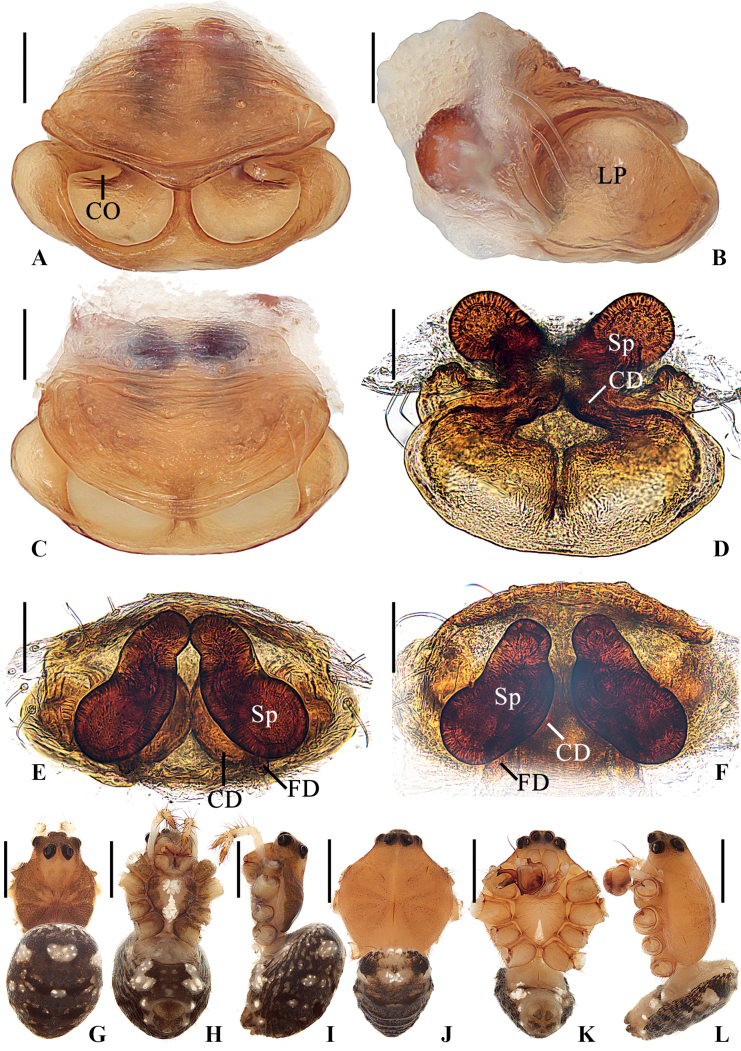
*Geaspinipes* C.L. Koch, 1843 **A–E, G–I**TRU-Araneidae-274 **F**TRU-Araneidae-276 **J–L**TRU-Araneidae-272 **A** epigyne, ventral view **B** ibid., lateral view **C** ibid., anterior view **D** vulva, posterior view **E** ibid., dorsal view **F** ibid., dorsal view **G, J** habitus, dorsal view **H, K** ibid., ventral view **I, L** ibid., lateral view. Scale bars: 0.1 mm (**A–F**); 1 mm (**G–L**). Abbreviations: CD copulatory duct, CO copulatory opening, FD fertilization duct, LP lateral plate, Sp spermatheca.

**Figure 4.**
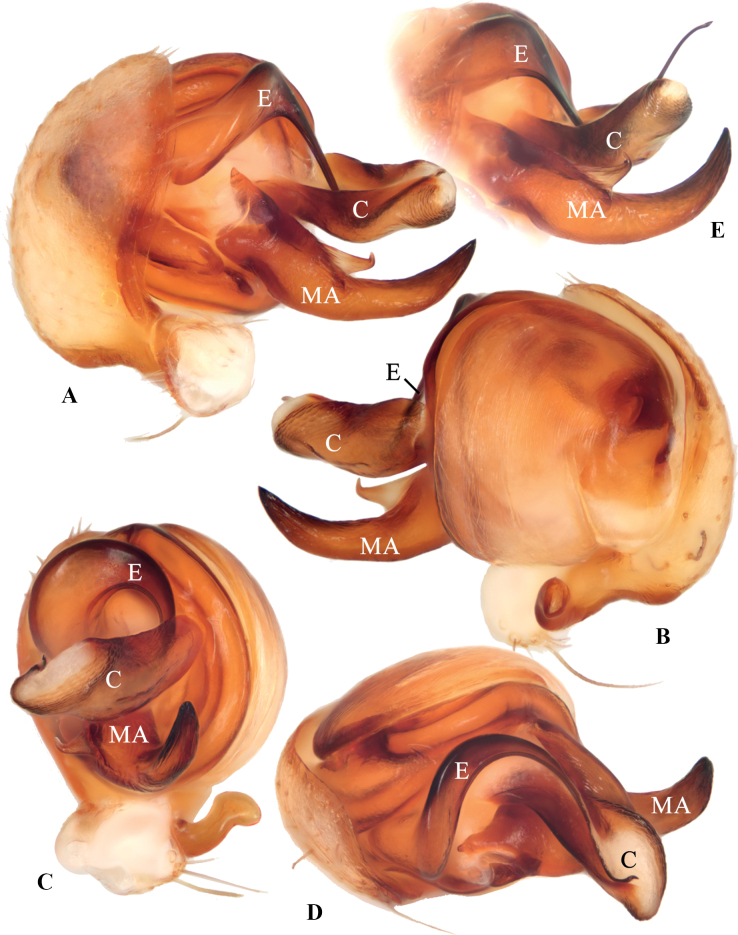
*Geaspinipes* C.L. Koch, 1843 **A–D**TRU-Araneidae-272 **E**TRU-Araneidae-279 **A** pedipalp, prolateral view **B** ibid., retrolateral view **C** ibid., ventral view **D** ibid., apical view **E** part of pedipalp (show the unbroken tip of embolus), prolateral view. Scale bars: 0.1 mm. Abbreviations: C conductor, E embolus, MA median apophysis.

**Figure 5.**
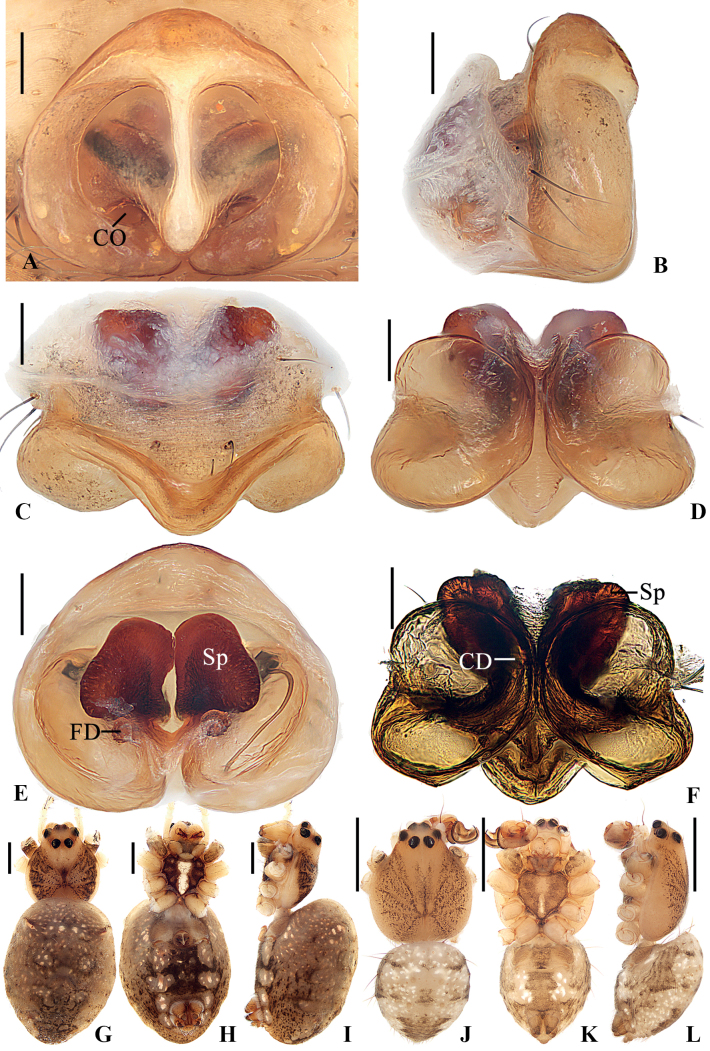
*Geasubarmata* Thorell, 1890 **A–I**TRU-Araneidae-289 **J–L**TRU-Araneidae-288 **A** epigyne, ventral view **B** ibid., lateral view **C** ibid., anterior view **D** ibid., posterior view **E** vulva, dorsal view **F** ibid., posterior view **G, J** habitus, dorsal view **H, K** ibid., ventral view **I, L** ibid., lateral view. Scale bars: 0.1 mm (**A–F**); 1 mm (**G–L**). Abbreviations: CD copulatory duct, CO copulatory opening, FD fertilization duct, Sp spermatheca.

The new species resembles *G.spinipes* in appearance and genitalia structures, but it can be distinguished as follows: 1) median apophysis not exceeding the conductor in prolateral view and retrolateral view (Fig. 2A, B) vs exceeding the conductor (Fig. 4A, B); 2) visible part of embolus curled about 90° in ventral view (Fig. 2C) vs about 180° (Fig. 4C); 3) conductor shorter, extending ventrally and not exceeding prolateral margin of pedipalp in ventral view (Fig. 2C) vs longer, extending ventro-prolaterally and the tip exceeding the prolateral margin of pedipalp (Fig. 4C); 4) copulatory openings situated on inner edges of the depressions (Fig. 1A) vs on anterior lateral edges (Fig. 3A); 5) lateral epigynal plates not covering the anterior rim in lateral view (Fig. 1B) vs covering the anterior rim (Fig. 3B); and 6) female carapace lacking dark brown patches (Fig. 1E, G) vs having dark brown patches (Fig. 3G, I).

##### Description.

**Male** (holotype, Figs 1H–J, 2, 7A–D). Total length 3.80. Carapace 2.25 long, 1.90 wide. Abdomen 1.90 long, 1.45 wide. Clypeus 0.05 high. Eye sizes and interdistances: AME 0.15, ALE 0.08, PME 0.15, PLE 0.15, AME–AME 0.13, AME–ALE 0.03, PME–PME 0.20, PME–PLE 0.18, MOA length 0.53, anterior width 0.38, posterior width 0.45. Leg measurements: I 8.85 (2.75, 2.65, 2.40, 1.05), II 8.35 (2.60, 2.45, 2.30, 1.00), III 4.55 (1.55, 1.30, 1.10, 0.60), IV 7.05 (2.35, 2.00, 1.90, 0.80). Carapace yellow, with inconspicuous gray patches in thoracic region; base of eyes black. Cervical groove inconspicuous; fovea longitudinal. Chelicerae yellow, with four promarginal and three retromarginal teeth. Endites wider than long, yellow, with very narrow, dark anterior edges. Labium triangular, yellow. Sternum cordiform, yellow, with a wedge-shaped white patch posteriorly. Legs yellow to yellowish brown, with inconspicuous annuli; femur I with 12 macrosetae; tibia I with 12 macrosetae; tibia II with seven macrosetae; tibia III with seven macrosetae; tibia IV with seven macrosetae. Abdomen shield-shaped, ~1.31× longer than wide, grayish yellow, dorsal with a pair of dark brown patches anterolaterally and a dark brown folium posteriorly. Venter abdomen yellow with gray patches. Spinnerets yellow with gray tip.

***Pedipalp*** (Fig. 2): paracybium fingerlike; median apophysis bifurcated; dorsal ramus weaker than the ventral one; embolus thick at base, twisted and tapered into a fine tip; conductor membranous, curled, about 2× longer than wide in retrolateral view.

**Female** (paratype TRU-Araneidae-269, Fig. 1A–G). Total length 5.05. Carapace 2.35 long, 2.00 wide. Abdomen 3.00 long, 2.35 wide. Clypeus 0.08 high. Eye sizes and interdistances: AME 0.15, ALE 0.08, PME 0.18, PLE 0.18, AME–AME 0.15, AME–ALE 0.03, PME–PME 0.33, PME–PLE 0.35, MOA length 0.58, anterior width 0.43, posterior width 0.65. Leg measurements: I 8.45 (2.45, 2.80, 2.20, 1.00), II 8.35 (2.45, 2.75, 2.15, 1.00), III 5.10 (1.65, 1.60, 1.15, 0.70), IV 8.00 (2.60, 2.55, 2.00, 0.85). Habitus similar to that of male, but abdomen with a pair of low anterolateral humps and sternum with a throughout paler patch.

***Epigyne*** (Fig. 1A–D): ~1.36× wider than long in ventral view, with a distinct median septum separating two depressions; copulatory openings situated on inner edges of the depressions; copulatory ducts widest at the beginning part, a bit longer than spermatheca; spermathecae almost S-shaped in dorsal view, not touching.

##### Variation.

Total length: ♀ 5.05–6.70 (*n* = 3).

##### Distribution.

China (Yunnan).

#### ﻿ Gea spinipes

Taxon classificationAnimaliaAraneaeAraneidae

C.L. Koch, 1843

B020C8A1-E73B-59D5-9129-5FD19F9ECC8E

Figs 3 , 4 , 7E–H , 8

Gea spinipes C.L. Koch 1843: 101, fig. 823; Yin et al. 1989: 67, fig. 7A–C; Chang and Chang 1997: 83, figs 1–4; Yin et al. 1997: 90, fig. 21a–f; Song et al. 1999: 282, fig. 169B–D. (type material not examined).

##### Materials examined.

China – Guangxi Zhuang Autonomous Region • 1♂; Beihai City, Yinhai District, Yajishan Forestry Station; 21°35.37'N, 109°18.41'E; ca 30 m elev.; 12.VIII.2017; X.Q. Mi et al. leg.; TRU-Araneidae-272 • 1♂; Fangchenggang City, Shangsi County, Shiwandashan National Forestry Park; 21°53.87'N, 107°54.26'E; ca 370 m elev.; 14.VIII.2017; X.Q. Mi et al. leg.; TRU-Araneidae-273 • 1♂; Beihai City, Tieshangang District, Xinggang Township, Xiaomatou Village, Caobiaotang; 21°33.11'N, 109°29.22'E; ca 10 m elev.; 4.XII.2018, X.Q. Mi et al. leg.; TRU-Araneidae-275 • 2♀♀; Chongzuo City, Jiangzhou District, Zuozhou Township, Guanghe Village; 22°34.72'N, 107°24.94'E; ca 160 m elev.; 4.VII.2019; C. Wang et al. leg.; TRU-Araneidae-276–277. – Guangdong Province • 1♀; Maoming City, Xinyi City, Dawuling Natural Reseve; 22°17.05'N, 111°10.87'E; ca 700 m elev.; 2.XII.2018, X.Q. Mi et al. leg.; TRU-Araneidae-274. – Hainan Province • 1♀;Wuzhishan City, A’tuoling; 18°50.17'N, 109°30.61'E; ca 790 m elev.; 9.VIII.2020 X.Q. Mi et al. leg.; TRU-Araneidae-278 • 1♀; Wuzhishan City, Shuiman Township, around Yataiyulin Hotel; 18°54.37'N, 109°40.70'E; ca 750 m elev.; 11.VIII.2020; X.Q. Mi et al. leg.; TRU-Araneidae-279 • 1♀; Dongfang City, Gancheng Township, Tuotou Village; 18°50.57'N, 108°50.87'E; ca 110 m elev.; 29.VII.2023; X.Q. Mi et al. leg.; TRU-Araneidae-280 • 1♂; Dongfang City, Gancheng Township, Tuotou Village, Shi’anlao; 18°50.56′N, 108°50.72′E; ca 110 m elev.; 30.VII.2023; X.Q. Mi et al. leg.; TRU-Araneidae-281 • 1♂1♀; Lingshui Li Autonomous County, Diaoluoshan National Nature Reserve, Popular Science Base; 18°40.25'N, 109°53.66'E; ca 490 m elev.; 26.VII.2023; C. Wang et al. leg.; TRU-Araneidae-282–283 • 1♂; Lingshui Li Autonomous County, Diaoluoshan National Nature Reserve, Shidai Village, Heliuling; 18°47.55'N, 109°44.03'E; ca 610 m elev.; 27.VII.2023; C. Wang et al. leg.; TRU-Araneidae-284 • 1♂; Lingshui Li Autonomous County, Diaoluoshan National Nature Reserve, Houshan; 18°43.57'N, 109°52.04'E; ca 930 m elev.; 28.VII.2023; C. Wang et al. leg.; TRU-Araneidae-285 • 1♂; Changjiang Li Autonomous County, Qicha Township, Bawangling National Nature Reserve, Dongyi Forest Station; 19°7.23'N, 109°7.64'E; ca 490 m elev.; 3.VIII.2023; X.Q. Mi et al. Leg; TRU-Araneidae-286 • 1♀; Baoting Li and Miao Autonomous County, Maogan Township, X124 roadside; 18°39.32'N, 109°32.45'E; ca 530 m elev.; 4.VIII.2023; C. Wang et al. leg; TRU-Araneidae-287.

##### Diagnosis.

See the Diagnosis of *G.jingdong* Mi, Wang & Gan, sp. nov.

##### Description.

**Male** (Figs 3J–L, 4, 7E–H). Total length 3.25. Carapace 1.95 long, 1.70 wide. Abdomen 1.85 long, 1.20 wide. Clypeus 0.05 high. Eye sizes and interdistances: AME 0.13, ALE 0.08, PME 0.13, PLE 0.13, AME–AME 0.13, AME–ALE 0.03, PME–PME 0.20, PME–PLE 0.23, MOA length 0.53, anterior width 0.38, posterior width 0.43. Leg measurements: I 8.55 (2.50, 2.60, 2.40, 1.05), II 7.90 (2.35, 2.30, 2.25, 1.00), III 4.65 (1.55, 1.30, 1.15, 0.65), IV 7.20 (2.35, 2.00, 2.00, 0.85). Carapace yellow, with inconspicuous, radial, dark patches; eyes with dark base. Cervical groove inconspicuous; fovea longitudinal. Chelicerae yellow, with four promarginal and three retromarginal teeth. Endites wider than long, yellow, with very narrow, dark anterior edges. Labium triangular, yellow. Sternum cordiform, yellow, with a white, wedge-shaped patch. Legs yellow to yellowish brown; legs III and IV with dark annuli; femur I with 11 macrosetae; tibia I with 13 macrosetae; tibia II with 13 macrosetae; tibia III with seven macrosetae; tibia IV with 11 macrosetae. Abdomen shield-shaped, ~1.54× longer than wide; dorsum dark, with two white spots anteriorly. Venter abdomen yellow, with white patches. Spinnerets yellow with gray tip.

***Pedipalp*** (Fig. 4): paracybium fingerlike; median apophysis bifurcated, dorsal ramus weaker, ventral ramus extremely long, exceeding length of conductor in prolateral and retrolateral view; embolus stout at base, twisted approximately 360° and tapering into a fine tip; conductor prominent, curled bilaterally.

**Female** (Fig. 3A–I). Total length 3.65. Carapace 2.05 long, 1.60 wide. Abdomen 2.40 long, 1.75 wide. Clypeus 0.05 high. Eye sizes and interdistances: AME 0.18, ALE 0.08, PME 0.18, PLE 0.18, AME–AME 0.10, AME–ALE 0.05, PME–PME 0.25, PME–PLE 0.35, MOA length 0.65, anterior width 0.38, posterior width 0.55. Leg measurements: I 6.60 (2.00, 2.05, 1.75, 0.80), II 6.50 (2.05, 2.00, 1.70, 0.75), III 4.05 (1.30, 1.25, 0.95, 0.55), IV 6.30 (2.10, 2.00, 1.60, 0.60). Habitus similar to that of male but with darker patches on thoracic region.

***Epigyne*** (Fig. 4A–F): ~1.3× wider than long in ventral view, with a distinct median septum separating two depressions in ventral view; copulatory openings situated on anterolateral edges of depressions; copulatory ducts twisted, a bit longer than spermatheca; spermathecae elongate kidney-shaped, touching or nearly touching at midline.

##### Variation.

Total length: ♂ 3.25–4.00 (*n* = 8); ♀ 3.65–6.90 (*n* = 8). Tip of embolus always broken.

##### Distribution.

China (Guangdong, Guangxi, Guizhou, Hainan, Taiwan, Yunnan), Pakistan, India, Indonesia, Malaysia, Myanmar, and Singapore.

##### Comment.

*Geaspinipes* is widely distributed from Pakistan to Indonesia, and shows some differences in epigynal structure of specimens collected from different sites (Levi 1983: figs 362–370) and that may indicate they are not conspecific. So, further taxonomic study about this species is necessary, especially getting more male specimens from different sites. *G.zaragosa* described by Barrion and Litsinger (1995) is similar to *G.spinipes* both in habitus and genitalia structures, but no detailed diagnosis was provided. Judging from the illustrations, *G.zaragosa* Barrion & Litsinger, 1995 is probably synonymized with the former.

#### ﻿ Gea subarmata

Taxon classificationAnimaliaAraneaeAraneidae

Thorell, 1890

4D4D0E4C-721C-583D-AB73-38AC07058C3E

**Figure 6.**
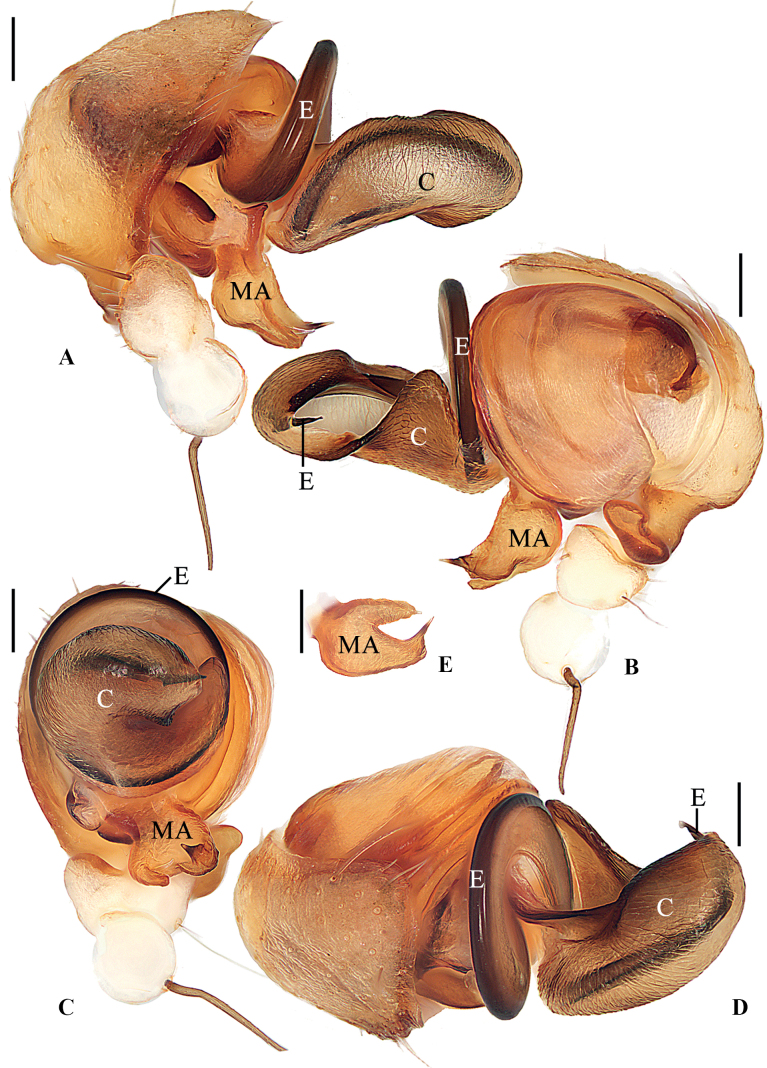
*Geasubarmata* Thorell, 1890 TRU-Araneidae-288 **A** pedipalp, prolateral view **B** ibid., retrolateral view **C** ibid., ventral view **D** ibid., apical view **E** median apophysis, dorsal view. Scale bars: 0.1 mm. Abbreviations: C conductor, E embolus, MA median apophysis.

**Figure 7.**
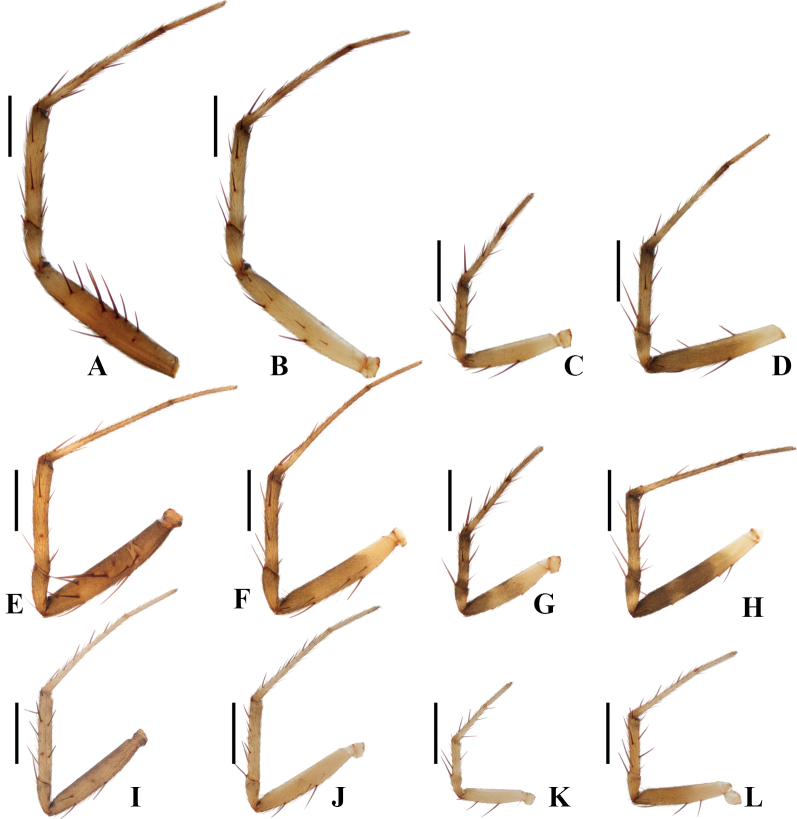
Legs of *Gea* spp., prolateral view (some macroseta fell out from the original positions) **A–D***Geajingdong* Mi, Wang & Gan, sp. nov. holotype **E–H***Geaspinipes* C.L. Koch, 1843 TRU-Araneidae-272 **I–L***Geasubarmata* Thorell, 1890 TRU-Araneidae-288 **A, E, I** legs I **B, F, J** legs II **C, G, K** legs III **D, H, L** legs IV. Scale bars: 1 mm.

**Figure 8.**
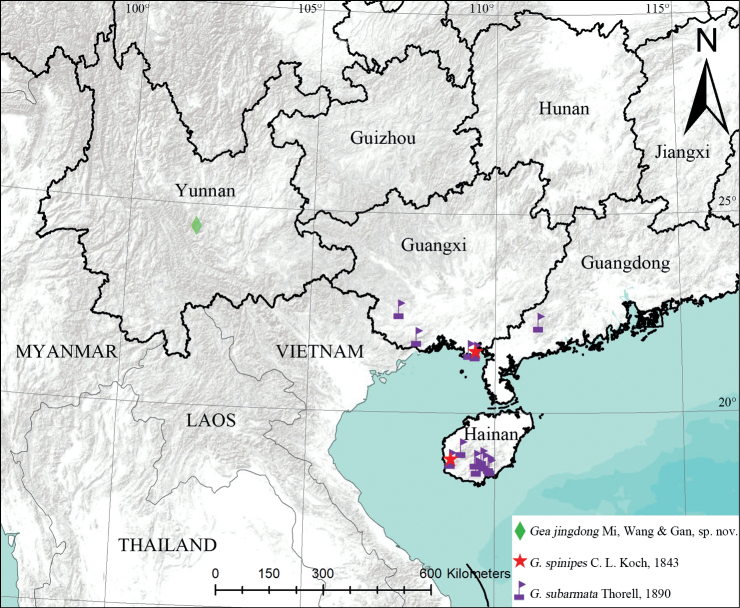
Distribution in China of the examined specimens.

Figs 5 , 6 , 7I–L , 8

Gea subarmata Thorell, 1890: 101; Levi 1983: 323, figs 350–354; Okuma et al. 1993: 21, fig. 16A, B (type material not examined).

##### Materials examined.

China – Guangxi Zhuang Autonomous Region • 1♂; Beihai City, Tieshangang District, Xinggang Township, Xiaomatou Village, Caobiaotang; 21°33.11'N, 109°29.22'E; ca 10 m elev.; 4.XII.2018; X.Q. Mi et al. leg.; TRU-Araneidae-288. – Hainan Province • 2♀♀; Dongfang City, Gancheng Township, Tuotou Village, Shi’anlao; 18°50.56′N, 108°50.72′E; ca 110 m elev.; 30.VII.2023; X.Q. Mi et al. leg.; TRU-Araneidae-289–290.

##### Diagnosis.

Females differ from those of congeneric species by the circular epigynum frame in ventral view (Fig. 5A) and bean-shaped spermathecae (Fig. 5E); males resembles *G.eff* Levi, 1983 in having similar pedipalp structures, but differs in: 1) dorsal ramus of the median apophysis tapered (Fig. 6A, C) vs slender (Levi 1983: figs 360, 361); 2) dorsal ramus of the median apophysis shorter than ventral ramus (Fig. 6E) vs about equal length (Levi 1983: fig. 360); and 3) conductor curled into a triangular dorsal fin in retrolateral view (Fig. 6B) vs lacking a triangular dorsal fin (Levi 1983: fig. 361).

##### Description.

**Male** (TRU-Araneidae-288, Figs 5J–L, 6, 7I–L). Total length 3.00. Carapace 1.65 long, 1.35 wide. Abdomen 1.65 long, 1.20 wide. Clypeus 0.10 high. Eye sizes and interdistances: AME 0.10, ALE 0.05, PME 0.10, PLE 0.10, AME–AME 0.10, AME–ALE 0.03, PME–PME 0.18, PME–PLE 0.20, MOA length 0.43, anterior width 0.33, posterior width 0.33. Leg measurements: I 6.55 (1.85, 2.00, 1.80, 0.90), II 6.05 (1.75, 1.80, 1.65, 0.85), III 3.20 (1.05, 0.95, 0.70, 0.50), IV 4.90 (1.60, 1.40, 1.25, 0.65). Carapace yellow, with dark patches on thoracic region. Cervical groove inconspicuous; fovea longitudinal. Chelicerae yellow, with four promarginal and three retromarginal teeth. Endites wider than long, grayish yellow, with very narrow, dark anterior edge. Labium triangular, grayish yellow, with paler at tip. Sternum cordiform, yellowish brown, with a paler longitudinal patch. Legs yellow without annuli; femur I with five macrosetae; tibia I with nine macrosetae; tibia II with eight macrosetae, tibia III with four macrosetae; tibia IV with nine macrosetae. Abdomen shield-shaped, ~1.38× longer than wide; dorsum whitish yellow, with a pair of narrow, grayish-brown patches anterolaterally and a grayish-brown folium posteriorly. Venter abdomen whitish yellow, with grayish-brown patches. Spinnerets yellowish brown.

***Pedipalp*** (Fig. 6): paracybium flattened, fingerlike; median apophysis bifurcate; dorsal ramus about equal length to ventral ramus; ventral ramus with a distal spur; embolus extremely long, twisted more than 360°; conductor prominent, membranous, wrapped around distal half of embolus.

**Female** (TRU-Araneidae-289, Fig. 5A–I). Total length 5.90. Carapace 2.30 long, 2.00 wide. Abdomen 3.80 long, 3.00 wide. Clypeus 0.10 high. Eye sizes and interdistances: AME 0.13, ALE 0.08, PME 0.13, PLE 0.13, AME–AME 0.25, AME–ALE 0.08, PME–PME 0.30, PME–PLE 0.35, MOA length 0.70, anterior width 0.50, posterior width 0.55. Leg measurements: I 8.30 (2.40, 2.75, 2.20, 0.95), II 7.95 (2.40, 2.65, 2.00, 0.90), III 4.85 (1.55, 1.55, 1.10, 0.65), IV 7.60 (2.40, 2.50, 1.95, 0.75). Habitus similar to that of male but abdomen with a pair of low anterolateral humps, thoracic region, sternum and abdomen a bit darker, and paler patch on sternum more obvious.

***Epigyne*** (Fig. 5A–F) ~1.2× wider than long, with circular frame and a long median septum separating two depressions in ventral view; copulatory openings located at posterior edges of depressions; copulatory ducts twisted into a C-shape, a bit longer than spermatheca; spermathecae bean-shaped, touching at midline.

##### Variation.

Total length: ♀ 5.60–5.90 (*n* = 2).

##### Distribution.

China (Hainan, Guangxi), Bangladesh, India, Indonesia, Japan, Malaysia, Myanmar, New Guinea, Philippines, and Singapore.

##### Comment.

Male of *G.subarmata* described by Kulczyński (1911) is the male of *G.eff* Levi, 1983 as Levi (1983) proposed.

## Supplementary Material

XML Treatment for Gea

XML Treatment for Gea jingdong

XML Treatment for Gea spinipes

XML Treatment for Gea subarmata

## ﻿Additional information

### Author ORCIDs

Xiaoqi Mi https://orcid.org/0000-0003-1744-3855

### Data availability

All of the data that support the findings of this study are available in the main text.
